# Gender-concordant identity documents and mental health among transgender adults in the USA: a cross-sectional study

**DOI:** 10.1016/S2468-2667(20)30032-3

**Published:** 2020-03-17

**Authors:** Ayden I Scheim, Amaya G Perez-Brumer, Greta R Bauer

**Affiliations:** Department of Medicine, University of California, San Diego, La Jolla, CA, USA; Department of Epidemiology and Biostatistics, Dornsife School of Public Health, Drexel University, Philadelphia, PA, USA; Division of Social and Behavioural Sciences, Dalla Lana School of Public Health, University of Toronto, Toronto, ON, Canada; Department of Epidemiology and Biostatistics, Schulich School of Medicine and Dentistry, Western University, London, ON, Canada

## Abstract

**Background:**

Transgender (trans) people experience profound mental health disparities compared with the general population, attributable in part to the psychological effects of gender non-affirmation. Despite the barriers to legal gender affirmation for trans people, little is known about its association with mental health. We therefore sought to determine whether having gender-concordant identity documents (IDs) is associated with mental health among trans adults in the USA. We hypothesised that having an ID that reflects one’s preferred name and gender marker would be associated with reduced psychological distress and suicide risk.

**Methods:**

In this cross-sectional observational study, we obtained data from the 2015 US Transgender Survey, the largest cross-sectional survey of trans adults in the USA, with 27 715 participants. Eligible participants were adults (≥18 years), residing in a US state, territory, or overseas US military base; and considered themselves transgender, trans, genderqueer, non-binary, or similar. We excluded participants not living day-to-day in a different gender to the sex they were assigned at birth, participants who identified as crossdressers, and those missing data. The primary exposure of interest was whether all or some (*vs* none) of a respondent’s IDs reflected their preferred name and gender marker. We examined associations with psychological distress (measured with the Kessler 6 scale) and suicide ideation, planning, and attempts in the past year, which we analysed using linear and modified Poisson regression models to examine associations with respondents’ IDs.

**Findings:**

Of 22 286 respondents included in our analytic sample, 10 288 (weighted percentage 45·1%) had their preferred name and gender marker on none, 9666 (44·2%) on some, and 2332 (10·7%) on all of their IDs. Compared with those with no gender-concordant ID, respondents for whom all IDs were concordant had lower prevalence of serious psychological distress (adjusted prevalence ratio 0·68, 95% CI 0·61–0·76), suicidal ideation (0·78, 0·72–0·85), and suicide planning (0·75, 0·64–0·87), adjusting for potential confounders. Having some versus no concordant ID was generally associated with smaller reductions in distress and suicidality. Gender-concordant ID was not associated with suicide attempts (eg, adjusted prevalence ratio for all *vs* no IDs was 0·92, 95% CI 0·68–1·24).

**Interpretation:**

Possession of gender-concordant IDs might improve mental health among trans persons. Gender recognition policies should be considered structural determinants of transgender health.

## Introduction

In the USA, 0·6% of adults, or 1·4 million people, are estimated to identify as transgender (ie, to have a gender identity that differs from the sex they were assigned at birth, which can fall outside the gender binary).^1^ According to the 2015 US Transgender Survey,^2^ only 11% of transgender (trans) people have their preferred name and gender marker on all identity documents (IDs) and official records. Among those whose IDs were not congruent with their gender presentation, a third experienced denied access to services, harassment, or violence, or all three.^2^ Despite these inequities, there is scant evidence concerning the health effects of legal gender recognition for trans people. IDs are required to obtain key health-promoting resources such as health care, housing, education, and employment.^3,4^ Moreover, they are required for immigration, travel, citizenship verification, security clearances, social service applications, and other major structural access points, as well as in daily activities such as socialising, purchasing items, and engaging in recreational activities.^2^ IDs can thus be conceptualised as a structural determinant of health linked to “socioeconomic-political context [as they are] structural mechanisms generating social stratification and the resulting socio economic position of individuals”.^5^

Research has underscored the prevalence of mental health disparities and psychological distress among trans people as compared with the general population. For example, the US Centers for Disease Control and Prevention’s (CDC) Behavioral Risk Factor Surveillance System (BRFSS) survey indicates a higher prevalence of poor mental health in the past 30 days among trans adults than in cisgender adults.^6^ The prevalence of clinical depression in trans adults is estimated to be over 50%,^7^ compared with an estimated 30% lifetime prevalence among the US general population.^8^ Furthermore, the lifetime prevalence of suicide attempts among trans adults is estimated to be 32–41%,^9,10^ compared with less than 9% among the general population^11^ and about 10–20% among lesbian, gay, and bisexual (LGB) adults.^12,13^

Research has documented numerous, intersecting stressors that are faced by transgender populations, due to societal stigma, which are associated with mental health disparities.^3,6,7^ To understand the adverse health effects, the minority stress model, a conceptual framework originally developed to explain mental health disparities in LGB populations,^14,15^ has been extended to trans populations.^9,16,17^ The minority stress framework examines the adverse mental health effects of stressors that result from dis crimination, rejection, and victimisation; the gender minority stress model further considers stress that is related to the non-affirmation of gender identity.^16^ Gender affirmation, defined as an interpersonal and social process of recognising and actualising one’s gender identity, might improve mental health within a gender minority stress framework via the direct effects of affirmation on wellbeing and through reduced exposure to other minority stressors (eg, discrimination and violence).^4,18–20^ Changing one’s name and gender marker on IDs such as birth certificates, passports, and driver’s licenses can be a crucial step in legal and social gender affirmation.

Yet, the process can range from difficult to impossible because much variation exists in the ID change process; for example, in most US states, updating a name on any government-issued ID first requires a court-ordered name change.^21^ Gender reclassification policies exist in most states, although not all, and require medical letters or affidavits to validate reclassification requests; they can require surgeries regardless of individual needs or contraindications. In most jurisdictions, gender markers reflecting non-binary gender identity (eg, an X marker) are not yet available.^21^ Although data is scarce in the USA context on the relationship between gender-concordant ID and wellbeing for trans adults, among trans youth, chosen name use has been associated with reduced depression, suicidal ideation, and suicidal behavior.^22^ A Canadian study, which used respondent-driven sampling methods among trans persons aged 16 years or older, documented that having at least one legal ID with a gender marker corresponding to a person’s lived gender was associated with reductions in suicidal ideation and attempts in the past year.^23^

To better understand the mental health effects of legal gender recognition, we assessed the relationship between gender-concordant ID and psychological distress and suicidality among trans adults in the USA. We hypothesised that having an ID that reflects one’s preferred name and gender marker would be associated with reduced psychological distress and suicide risk, including ideation, planning, and attempts. In addition, among respondents who had no ID with their preferred gender marker, we describe the reasons why they had not changed the marker.

## Methods

### Study design and participants

This study is a secondary data analysis of the 2015 US Transgender Survey, a cross-sectional survey of trans adults conducted by the US National Center for Transgender Equality. Eligible participants were aged 18 years or older; resided in a US state, territory, or overseas US military base; and considered themselves transgender, trans, genderqueer, non-binary (neither male nor female), or a similar identity. Data were collected in English and Spanish through a self-administered online survey in 2015. Participants were recruited through outreach to transgender and LGBT organisations, health centres, support groups, and online communities. Recruitment and data collection methods are further detailed in the study report.^2^ The study sample included 27 715 respondents from all 50 US states, the District of Columbia, three US territories (American Samoa, Guam, and Puerto Rico), and US military bases overseas. No response rate could be calculated because of the absence of a sampling frame. The US Transgender Survey was approved by the Institutional Review Board (IRB) at the University of California Los Angeles, USA, and this secondary analysis was deemed exempt from IRB review by the University of California San Diego Institutional Review Board (#181512XX).

Because name change and legal gender recognition policies are most relevant to trans people living day-today in a gender that differs from the sex they were assigned at birth, we excluded from this analysis participants who were not living full-time or part-time in a gender different from their assigned sex at birth (n=4344) and participants who identified as crossdressers (n=512). We also excluded 573 respondents who were missing data for the exposure or outcomes.

### Exposure

Our primary exposure of interest was whether respondents had gender-concordant IDs at the time of the study. Respondents were asked two separate questions about the name and gender marker on their IDs and records: “Thinking about how your name [gender] is listed on all of your IDs and records that list your name, such as your birth certificate, driver’s license, passport, etc. Which of the statements below is most true?” Respondents could indicate that all, some, or none “of my IDs and records list the name [gender] I prefer”. We coded these items into a single variable indicating whether all, some, or none of a respondent’s IDs and records reflected their preferred name and gender marker. The group with some gender-concordant IDs includes individuals for whom the name or gender marker that they prefer, or both, was reflected on some, but not all, IDs and records.

### Outcomes

Our primary outcomes of interest were psychological distress and suicidal ideation. Pyschological distress was measured with the Kessler 6 scale,^24^ which includes six items that assess the frequency of symptoms of non-specific psychological distress (eg, feeling hopeless or nervous) in the past 30 days. Scores range from 0 to 24, with higher scores indicating greater distress. We modelled psychological distress as both a continuous and dichotomous outcome, using the validated cut off of 13 points or greater to indicate serious psychological distress, which has shown good sensitivity and excellent specificity for diagnosed serious mental illness in population samples.^25^ Suicide risk during the past year was assessed by asking respondents “At any time in the past 12 months did you seriously think about trying to kill yourself?”. Those answering yes were then asked “Did you make any plans to kill yourself?” and “Did you try to kill yourself?” in the same time period. We forward-filled the planning and attempt variables (ie, we coded a respondent who had not considered suicide as not having planned or attempted suicide) to generate binary outcomes for suicide ideation, planning, and attempts for the full sample.

To consider potential confounders, a directed acyclic graph (appendix p 1) was developed based on our review of previous literature on psychological distress and suicidality among trans people. Considering the scarcity of research on gender-concordant IDs among trans people, we also considered temporality, plausibility, and background knowledge on ID policies in diagramming directed edges towards the exposure. A set of sociodemographic and social context variables were chosen for control of confounding on the basis of the directed acyclic graph. Social support was identified in our directed acyclic graph as a potential confounder requiring control, but no corresponding variable was available in the dataset.

We used standard US Transgender Survey recodes unless otherwise specified. The survey team coded gender (trans woman, trans man, non-binary assigned female, non-binary assigned male) by cross-classifying sex assigned at birth and gender identity; non-binary respondents were those who selected “non-binary/genderqueer” in the forced-choice gender question. Other demographic covariates included age (continuous), race and ethnicity (using the American Community Survey categories), education level (eg, high school or Bachelor’s degree), poverty (above *vs* at or near [101–124% of] the Census Bureau poverty threshold), US census region, whether the respondent was US-born, history of active military duty, family support for gender identity (eg, supportive, neutral, or unsupportive), and years living full-time in one’s felt gender (imputed as 0 for those living part-time in one gender and part-time in another). We coded functional disability as yes (*vs* no) if a respondent reported a disability impacting cognitive ability, activities of daily living, ability to perform errands, or walking. Considering that genital surgery is sometimes required to change administrative or legal gender, we created a four-level medical transition status variable, which included “not needed” (never had or wanted hormone therapy or surgery), “not begun” (wanted hormone therapy or surgery), had “hormones or non-genital surgery, or both”, and had “genital surgery”.

With respect to respondents’ reasons for not changing their gender marker, if a respondent indicated having no ID reflecting their preferred gender marker, they were asked to select the reasons why from a list. Potential reasons included a lack of suitable gender options, not being ready, or not being able to afford it.

### Statistical analysis

We examined associations between respondents’ IDs reflecting their preferred name and gender, and psychological distress and past-year suicide ideation, planning, and attempts. Analyses were performed in SAS version 9.4 (SAS Institute; Cary, NC, USA). Because of the small amount of missing data in the study sample (<0·5% for all variables except poverty status, for which 4·6% of the respondents included were missing), we did not use imputation methods for categorical variables. To avoid case loss, we used mean imputation for age (for one respondent with missing data) and years living in a person’s felt gender (for 58 respondents); the results of analyses excluding these participants did not differ substantially and are not presented. We began by estimating the descriptive statistics that were stratified by exposure status. We fit bivariable and multivariable linear regression models for the continuous psychological distress outcome. We confirmed the suitability of data for linear regression by assessing multicollinearity, normality of residuals, and homoscedasticity using a variance inflation factor, q-q plots, and plots of residuals versus fitted values.

For binary outcomes, we used modified Poisson regression models in PROC GENMOD with robust error variances to estimate prevalence ratios because the outcomes were common.^26^ Because policies related to changes in name and gender marker are generally distinct, we conducted supplementary analyses in which the preferred name and gender marker (on all, some, or no IDs) were split into separate exposures. Finally, among respondents who indicated that none of their IDs reflected their preferred gender marker, we calculated frequencies for the reasons that they provided.

For all analyses, we applied a survey weight developed by the US Transgender Survey researchers to weigh the sample to the ethnoracial and age distribution of the US population, because the sample was younger and less ethnoracially diverse than the general population. The weight also accounts for the over-representation of people aged 18 years in the sample. Weighting had a notable effect on the estimated ethnoracial composition of the sample, but exposure prevalence was similar across race and ethnicity groups. Regression results in a sensitivity analysis without the survey weight did not differ appreciably and so we present the weighted results herein.

### Role of the funding source

No funding was received for this analysis. The corresponding author had full access to all of the data in the study and had the final responsibility to submit for publication.

## Results

The analytic sample included 22 286 respondents living full-time or part-time in a gender different from that assigned at birth (table 1). 10 288 (weighted percentage 45·1%) of these respondents had no IDs with their preferred name and gender marker, 9666 (44·2%) had some concordant IDs, and 2332 (10·7%) had their preferred name or gender marker on all of their IDs. Lower unadjusted levels of psychological distress on the Kessler 6 scale were reported by trans adults with all concordant IDs (mean 7·0 [standard error (SE) 0·2]) or some concordant IDs (9·7 [0·1]) than by those with no concordant IDs (12·2 [0·1]; table 1). Among the individuals with all or some IDs with their preferred name and gender, fewer respondents reported suicidal ideation, suicide planning, or suicide attempts in the past year than did individuals with no concordant IDs (table 1).

Multivariable models were adjusted for age, gender, race and ethnicity, education, poverty, census region, US birth, functional disability, active military duty, medical transition status, family support for gender, and years spent living full-time in one’s gender. In the adjusted linear regression model, having all or some IDs reflecting the preferred name and gender was associated with reduced psychological distress, with a decrease of 1·92 points (95% CI 1·56–2·27) on the 24-point Kessler 6 scale for having all concordant IDs, and a decrease of 0·75 points (0·53–0·96) for having some concordant IDs (table 2). Similarly, respondents with some or all concordant IDs were less likely to meet the threshold for serious psychological distress (defined as a score of ≥13 on the Kessler scale).

In adjusted modified Poisson models, respondents for whom all IDs were concordant with their preferred name and gender had a lower prevalence of suicidal ideation (adjusted prevalence ratio [APR] 0·78; 95% CI 0·72–0·85) and suicide planning (APR 0·75; 0·64–0·87) than did those with no concordant IDs (table 3). Having some (*vs* no) concordant IDs was associated with small reductions in suicidal ideation (APR 0·95; 0·91–0·98) and suicide planning (APR 0·93; 0·86–1·00). Respondents with all or some concordant IDs were significantly less likely than those with none to attempt suicide in our bivariable analyses, but not after controlling for the set of potential confounders (all IDs *vs* none: APR 0·92; 0·68–1·24; some IDs *vs* none: APR 0·96; 0·84–1·11).

Associations between concordant IDs, psychological distress, and suicidality were similar when considering preferred name and gender marker separately, with generally smaller magnitudes for preferred name than for gender marker (appendix pp 2–3). Among the 14 370 (weighted percentage 63·8%) respondents who had their preferred gender marker on none of their IDs, common reasons for not having changed their gender markers included feeling that gender options do not fit their gender identity (5896 [40·3%]) and prohibitive cost (4874 [34·2%]; table 4). 3951 (26·7%) respondents believed they were not allowed to change their marker (eg, because additional medical treatment was required).

## Discussion

To our knowledge, this is the first study in the USA to quantitatively examine the relationship between gender-concordant identity documents and psychological distress and suicidality among trans adults. Our results show associations between having some or all gender-concordant IDs and better mental health among US trans adults, extending previous research.^23^ Underscoring that legal gender affirmation is a structural determinant of health for trans people, who already face inequities in health and access to health-care, our findings support the substantial global momentum for legislative change for gender recognition policies as a fundamental human right.^3^

We found that most trans people living in their felt gender did not have fully gender-concordant IDs, with only 10·7% indicating that all of their IDs reflected both their preferred name and gender marker. The reasons for not changing their gender markers included a lack of suitable gender options (ie, beyond male or female), cost, and perceived ineligibility. The findings previously published in the US Transgender Survey report^2^ indicate that cost is a key barrier to legal name changes; court filing fees are typically several hundred US dollars. The reasons indicated by participants corresponded closely with the differences observed in our analyses, wherein those with non-binary identities, poverty-level incomes, or without gender-affirming surgeries were overrepresented in the group with no concordant IDs. Corresponding to state-level variation in ID policies,^27^ we also observed geographical variation; for example, participants in western USA were more likely to have gender-concordant IDs, and those in the midwest were less likely.

Psychological distress and unadjusted prevalence of suicide ideation, planning, and attempts in the past year increased in a stepwise fashion when comparing respondents for whom all, some, or none of their IDs reflected their preferred name and gender marker. After controlling for potential confounders, having all concordant IDs was associated with a 32% reduction in serious psychological distress and 22–25% reduction in suicidal ideation and suicide planning compared with having none. However, associations between having some versus no concordant IDs and mental health outcomes were smaller (5–7% reduction in suicidal ideation and suicide planning). Gender-concordant ID status was not associated with suicide attempts. Correlates of suicidal ideation and attempts are known to differ across a range of populations; ideation-to-attempt suicidology frame works posit that dispositional characteristics, acquired capability, and access to means are determinants of suicide attempts.^28^ Nonetheless, our results indicate that not having gender-concordant IDs might contribute to serious mental distress (evidenced by suicidal ideation and planning, which in turn predict future attempts and deaths by suicide).^29^

Our findings differ somewhat from the sole study to have addressed a similar question, which found that having gender-concordant ID was associated with reductions in suicide attempts among trans people in Ontario, Canada.^23^ That study, however, focused more narrowly on the effect among trans people who had socially transitioned to live as men or women of having at least one legal ID with a gender marker corresponding to their lived gender. The Ontario study also examined predictors of attempts only among the subgroup with ideation and did not control for medical transition status, a key potential confounder in our analyses.

Having gender-concordant IDs could affect mental health through many pathways. IDs and records serve as proof of identity and citizenship and are required for access to health care and social institutions such as employment and banking, as well as for full participation in social life.^3^ The US Transgender Survey estimated that 32% of respondents who had presented an ID that did not match their gender presentation had a negative experience, including verbal harassment (25%), denial of service (16%), and assault (2%).^2^ The effects of such discrimination and violence on psychological distress and suicide risk are well documented.^10,17,30^ Anticipation of mistreatment due to gender non-concordant ID might also damage a person’s mental health through vigilance, anxiety, and avoidance of social participation.^3,20^

Additionally, possessing gender-concordant IDs might promote positive mental health by affirming one’s gender identity and increasing access to interpersonal gender affirmation in social interactions.^3,20^ Our results suggest that having only some gender-concordant IDs has less of an effect, being associated with relatively small reductions in psychological distress and suicidal ideation only. Notably, there was a low threshold to be included in the group having some concordant IDs; experiences could range from having changed one’s name only on a single ID or record to having changed both one’s name and gender marker on all but one ID or record. It is plausible that a gradient exists within this group, with less distress and suicidality among those for whom a greater proportion of IDs are gender concordant. However, the fact that results were similar for our supplementary analyses separating names and gender markers allays concerns that our results are unduly influenced by coding decisions for exposures. The outcomes might further vary by type of identity document or record. Passports, for example, influence international travel and any requirements for citizenship validation, whereas driver’s licenses serve as common everyday IDs. Social security records and school records have specific effects in employment and educational settings. Future research using the US Transgender Survey or other data sources could explore these nuances, as well as the mediators and moderators of relationships between gender-concordant IDs and mental health outcomes.

Our findings have various limitations, particularly the cross-sectional study design, which precludes causal interpretations. It is possible that psychological distress and suicidality preceded the exposure; in particular, psychological distress could make it more difficult to obtain gender-concordant IDs. Residual confounding also poses a threat to validity, whether by social support (included in our directed acyclic graph but unmeasured) or by variables that might have been incorrectly excluded from our directed acyclic graph (appendix p 1). Finally, the non-probability sampling method limits the generalisability of our findings. We weighted the sample to the age and ethnoracial distribution of the US population, but there are other notable differences between the sample and US population demographics (eg, 94% of respondents were US-born). Moreover, it remains unclear whether trans population demographics do, in fact, mirror the broader US population.

Despite these limitations, the data were derived from the largest sample of trans adults ever surveyed and our analytical design controlled for confounding using a directed acyclic graph. We have documented that the possession of gender-concordant IDs is associated with reduced psychological distress, suicidal ideation, and suicide planning among trans adults in the USA. These findings highlight the imperative to consider administrative and legal gender recognition in trans health research, and they reinforce calls for gender affirmation to be considered as a key social and structural determinant of trans people’s health. Policy changes to increase access to gender-concordant IDs should be considered as potential structural interventions to improve the mental health of trans populations. Such policies could reduce fees, administrative hurdles, and eligibility requirements, expand gender marker options, or remove gender markers entirely.

## Supplementary Material

Supplement

## Figures and Tables

**Table 1:** Characteristics stratified by identity document status

	Total respondents (n=22 286)	Amount of identity documents reflecting preferred name and gender		
		None (n=10 288)	Some (n=9666)	All (n=2332)

Age, years (mean [SE])	30·9 (0·1)	27·8 (0·1)	32·1 (0·2)	39·1 (0·4)
Gender				
Transgender woman	7948 (35·6%)	3389 (33·9%)	3476 (35·0%)	1083 (45·3%)
Transgender man	7235 (33·1%)	3092 (29·7%)	3331 (35·8%)	812 (36·8%)
Non-binary, assigned female	5800 (25·5%)	3213 (30·6%)	2285 (23·3%)	302 (12·6%)
Non-binary, assigned male	1303 (5·8%)	594 (5·8%)	574 (5·9%)	135 (5·3%)
Race and ethnicity				
Alaska Native or American Indian	277 (0·7%)	138 (0·7%)	118 (0·7%)	21 (0·5%)
Asian or Pacific islander	624 (5·0%)	282 (4·9%)	284 (5·3%)	58 (4·7%)
Biracial, multiracial, or not listed	1212 (2·3%)	583 (2·4%)	520 (2·3%)	109 (1·9%)
Black or African American	657 (13·0%)	260 (11·7%)	319 (14·0%)	78 (14·1%)
Latinx or Hispanic	1202 (16·7%)	615 (18·4%)	469 (15·3%)	118 (15·7%)
White, Middle Eastern, or north African	18 314 (62·3%)	8410 (61·8%)	7956 (62·5%)	1948 (63·0%)
Education				
Less than high school	730 (2·6%)	522 (3·7%)	169 (1·6%)	39 (1·8%)
High school	2723 (10·5%)	1730 (14·1%)	824 (7·8%)	169 (6·9%)
Some college or Associate’s degree	10 329 (49·2%)	5266 (55·0%)	4235 (46·0%)	828 (38·0%)
Bachelor’s degree or higher	8504 (37·7%)	2770 (27·2%)	4438 (44·7%)	1296 (53·3%)
Poverty				
Above poverty threshold	14 075 (62·3%)	5842 (55·4%)	6432 (66·1%)	1801 (75·1%)
At or near poverty threshold	7177 (33·4%)	3826 (39·1%)	2878 (30·3%)	473 (22·2%)
Missing data	1034 (4·3%)	620 (5·5%)	356 (3·5%)	58 (2·7%)
US census region				
No census region	48 (0·4%)	33 (0·6%)	12 (0·2%)	3 (0·2%)
Northeast	4553 (20·3%)	1892 (17·7%)	2177 (23·2%)	484 (19·5%)
Midwest	4597 (18·8%)	2246 (20·0%)	1943 (18·5%)	408 (14·7%)
South	6019 (28·8%)	3099 (31·6%)	2367 (26·7%)	553 (25·9%)
West	7069 (31·7%)	3018 (30·0%)	3167 (31·4%)	884 (39·6%)
Born in the USA	21 474 (94·3%)	9943 (94·7%)	9303 (94·1%)	2228 (93·6%)
Functional disability	8573 (37·5%)	4792 (45·6%)	3234 (32·6%)	547 (23·8%)
Active military duty	1928 (8·6%)	715 (7·0%)	918 (9·3%)	295 (12·6%)
Medical transition				
Not needed	1974 (8·6%)	724 (6·7%)	1000 (10·3%)	250 (10·2%)
Not begun	7539 (32·2%)	5688 (52·9%)	1696 (17·3%)	155 (6·6%)
Hormones or non-genital surgery, or both	11 185 (52·5%)	3777 (39·6%)	6201 (65·3%)	1207 (54·3%)
Genital surgery	1501 (6·2%)	60 (0·5%)	727 (6·6%)	714 (28·6%)
Missing data	87 (0·4%)	39 (0·4%)	42 (0·5%)	6 (0·3%)
Family supportive of gender				
Supportive	10 851 (48·8%)	4176 (40·5%)	5266 (54·1%)	1409 (61·9%)
Neutral	3745 (167%)	1865 (17·9%)	1582 (16·4%)	298 (13·1%)
Unsupportive	3325 (14·6%)	1640 (15·4%)	1395 (14·6%)	290 (11·3%)
Not applicable or missing data	4365 (19·8%)	2607 (26·1%)	1423 (14·8%)	335 (13·7%)
Years living in gender full-time (mean [SE])	4·1 (0·1)	2·1 (0·2)	5·1 (0·1)	8·5 (0·3)
Outcomes				
Psychological distress on Kessler 6 scale (mean [SE])	10·5 (0·1)	12·2 (0·1)	9·7 (0·1)	7·0 (0·2)
Serious psychological distress	8576 (38·4%)	5070 (49·3%)	3063 (32·2%)	443 (18·8%)
Suicidal ideation	10 964 (48·8%)	5993 (57·6%)	4259 (44·3%)	712 (30·0%)
Suicide planning	5560 (24·1%)	3201 (29·6%)	2043 (21·1%)	316 (13·7%)
Suicide attempt	1739 (7·6%)	1052 (9·6%)	591 (6·4%)	96 (4·3%)

Data are in n (%) unless otherwise stated. Frequencies (n) are unweighted; proportions (%) are weighted to the age and race and ethnicity distribution of the US population from the American Community Survey. SE=standard error. At or near poverty threshold defined as 101–124% of Census Bureau poverty threshold. Serious psychological distress defined as 13 points or more on the Kessler 6 scale.

**Table 2:** Associations between amount of gender-concordant identity documents and psychological distress

	Unadjusted *B* (95% CI)	Adjusted *B* (95% CI)	Unadjusted prevalence ratio (95% CI)	Adjusted prevalence ratio (95% CI)

None	..	..	1·00	1·00
Some	−1·88 (−2·37 to −1·38)	−0·75 (−0·96 to −0.53)	0·65 (0·62 to 0·69)	0·88 (0·84 to 0·93)
All	−5·24 (−5·91 to −4·58)	−1·92 (−2·27 to −1·56)	0·38 (0·34 to 0·43)	0·68 (0·61 to 0·76)

Unstandardised *B* shows the change in psychological distress in terms of points on the 24-point Kessler 6 scale. Adjusted values take into account participants’ age, gender, race and ethnicity, education level, poverty level, census region, whether they are US-born, whether they have a functional disability, whether they have served in active military duty, medical transition status, family support level, and years living full-time in their gender. Prevalence ratios are for serious psychological distress, defined as 13 points or more on the Kessler 6 scale.

**Table 3:** Associations between amount of gender-concordant identity documents and suicide risk

	Suicidal ideation		Suicide planning		Suicide attempt	
	Unadjusted prevalence ratio (95% CI)	Adjusted prevalence ratio (95% CI)	Unadjusted prevalence ratio (95% CI)	Adjusted prevalence ratio (95% CI)	Unadjusted prevalence ratio (95% CI)	Adjusted prevalence ratio (95% CI)

None	1·00	1·00	1·00	1·00	1·00	1·00
Some	0·77 (0·74–0·80)	0·95 (0·91–0·98)	0·71 (0·67–0·76)	0·93 (0·86–1·00)	0·67 (0·59–0·77)	0·96 (0·84–1·11)
All	0·52 (0·48–0·57)	0·78 (0·72–0·85)	0·46 (0·40–0·53)	0·75 (0·64–0·87)	0·45 (0·34–0·60)	0·92 (0·68–1·24)

Adjusted values take into account participants’ age, gender, race and ethnicity, education level, poverty level, census region, whether they are US-born, whether they have a functional disability, whether they have served in active military duty, medical transition status, family support level, and years living full-time in their gender.

**Table 4:** Reasons for not changing gender marker on identity documents

	Number of respondents (n=14370)

Gender options do not fit my gender identity	5896 (40·3%)
Have not tried yet	6445 (43·4%)
Request was denied	231 (1·6%)
Not ready	3894 (26·2%)
Cannot afford it	4874 (34·2%)
Do not know how	3915 (26·7%)
Believe I am not allowed	3951 (26·7%)
Worried I might not be able to get benefits or services	3607 (25·0%)
Worried it would out me	3208 (21·1%)

Data are in n (%). Frequencies (n) are unweighted; proportions (%) are weighted to the age and race and ethnicity distribution of the US population from the American Community Survey. Respondents could select all that apply.

## Research in context

### Evidence before this study

In November, 2019, we searched peer-reviewed publications and available grey literature with no date restrictions to assess the available evidence on gender-concordant identity documents (IDs) and mental health among transgender adults. Search terms included those related to gender identity (“transgender” or “trans*”) and identification documents (“ID”, “identification”, “identification document*”, and “legal document*”). We also did a subset of searches that included terms related to “mental health”, “depression”, and “suicide”, but given the scant available literature, we amplified the search. A single Canadian study found that having at least one ID concordant with a person’s lived gender was associated with lower prevalence of suicidal ideation and suicide attempts. A few studies have assessed the relationships between other forms of social and legal gender affirmation and mental health among transgender persons. For example, a 2018 study found an association between a person’s chosen name being used in social contexts and reduced mental health risks among transgender adolescents.

### Added value of this study

To our knowledge, this study is the first to assess the relationship between gender-concordant IDs and mental health among transgender people in the USA. It draws on the largest sample of transgender people ever surveyed, the 2015 US Transgender Survey. We build on the single previous study (from Canada) examining this question by considering both psychological distress and suicidality, with improved control for confounding.

### Implications of all the available evidence

For transgender people living day-to-day in a gender different from their assigned sex at birth, access to gender-concordant identity documents might reduce their psychological distress and suicidal ideation. The potential effects on suicide attempts are unclear; longitudinal research is needed on the determinants of suicidal ideation and suicide attempts and completion in transgender populations. Gender recognition policies, at the intersection of social and political processes related to health inequities, might be structural determinants of transgender mental health and should be evaluated for their public health impacts.
