# Supplementary material for: Gender-concordant identity documents and mental health among transgender adults in the USA: a cross-sectional study
Source: Lancet Public Health. Author manuscript; Available in PMC 2023 Feb 10. (PMC9912749; doi:10.1016/S2468-2667(20)30032-3)
Supplement: Supplement [file NIHMS1860610-supplement-Supplement.pdf]

THE LANCET
Public Health

Supplementary appendix

This appendix formed part of the original submission and has been peer reviewed. We post it as supplied by the authors.

Supplement to: Scheim AI, Perez-Brumer AG, Bauer GR. Gender-concordant identity documents and mental health among transgender adults in the USA: a cross-sectional study. *Lancet Public Health* 2019; published online March 16. http://dx.doi.org/10.1016/S2468-2667(20)30032-3.

Supplementary Figure 1: Directed Acyclic Graph

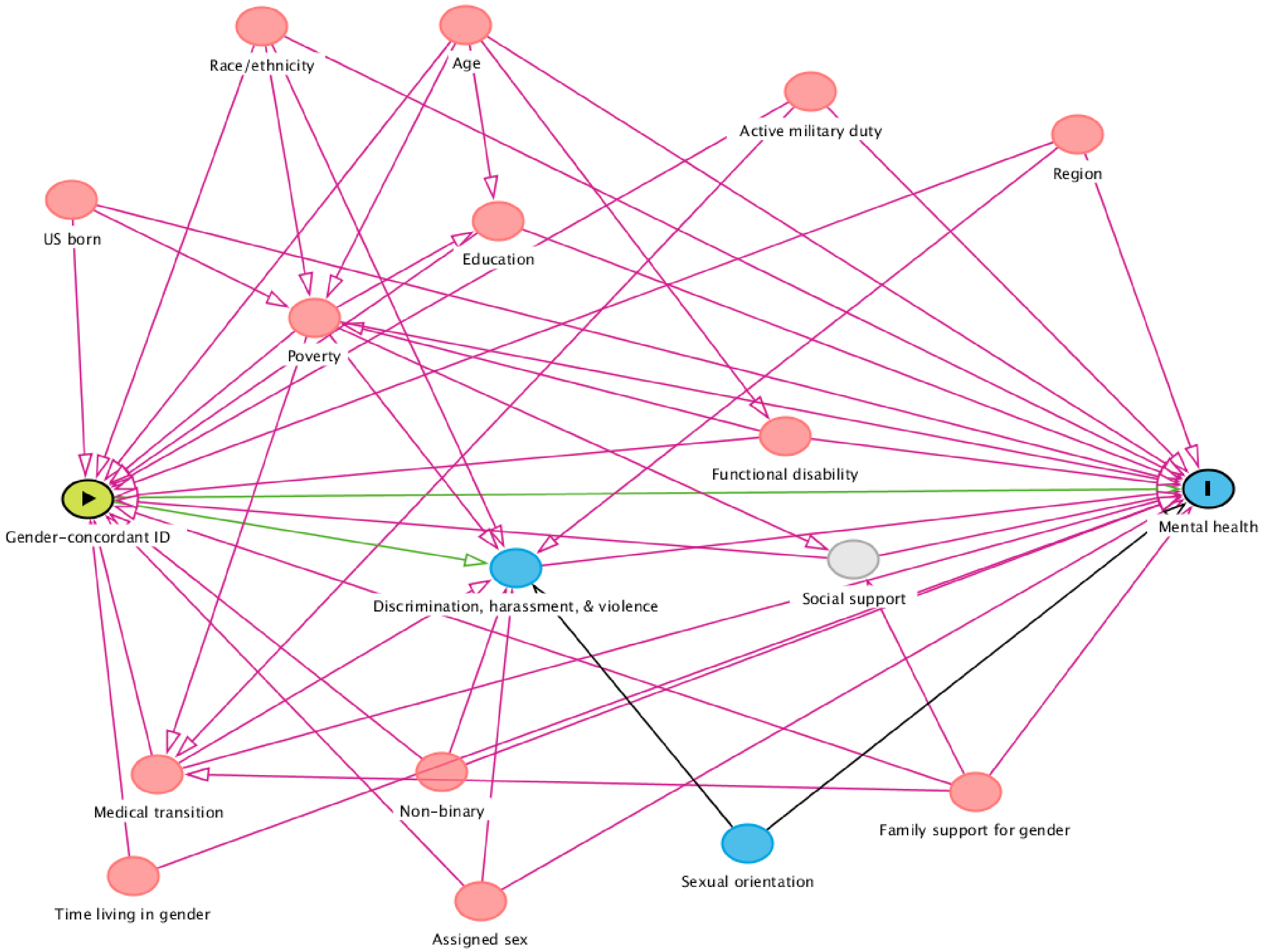

Software: Textor J, van der Zander B, Gilthorpe MK, Liskiewicz M, Ellison GTH. Robust causal inference using directed acyclic graphs: the R package 'dagitty'. *Int J Epidemiol* 2016; 45(6):1887-1894.

TABLE S1. Frequency of having identity documents with one's preferred name

Identity Documents Reflect Name	n (weighted%)
None	10615 (46.6)
Some	5579 (25.1)
All	6092 (28.3)

TABLE S2. Associations between having identity documents with one's preferred name and psychological distress

Identity Documents Reflect Name	Unadjusted *b* (95% CI)	Adjusted *b** (95% CI)	Unadjusted PR† (95% CI)	Adjusted PR*† (95% CI)
None	--	--	1.00	1.00
Some	-2.93 (-3.20, -2.65)	-0.66 (-0.90, -0.41)	0.61 (0.57, 0.65)	0.91 (0.85, 0.96)
All	-3.13 (-3.40, -2.85)	-1.16 (-1.40, -0.92)	0.59 (0.56, 0.63)	0.82 (0.77, 0.87)

* Adjusted for age, gender, race/ethnicity, education, poverty, census region, US-born, functional disability, active military duty, medical transition status, family support, and years living full-time in gender.

† Prevalence ratio for serious psychological distress (Kessler 6 ≧13)

TABLE S3. Associations between having identity documents with one's preferred name and suicide risk

Identity Documents Reflect Name	Suicidal ideation		Suicide plan		Suicide attempt	
	Unadjusted PR* (95% CI)	Adjusted PR*† (95% CI)	Unadjusted PR* (95% CI)	Adjusted PR*† (95% CI)	Unadjusted PR* (95% CI)	Adjusted PR*† (95% CI)
None	1.00	1.00	1.00	1.00	1.00	1.00
Some	0.74 (0.71, 0.78)	0.95 (0.91, 0.99)	0.74 (0.69, 0.80)	1.01 (0.92, 1.10)	0.68 (0.58, 0.80)	0.98 (0.83, 1.17)
All	0.70 (0.66, 0.73)	0.89 (0.85, 0.93)	0.60 (0.55, 0.65)	0.82 (0.75, 0.90)	0.55 (0.46, 0.65)	0.88 (0.73, 1.05)

* Prevalence Ratio

† Adjusted for age, gender, race/ethnicity, education, poverty, census region, US-born, functional disability, active military duty, medical transition status, family support, and years living full-time in gender.

TABLE S4. Frequency of having identity documents with one's preferred gender marker

Identity Documents Reflect Gender	n (weighted%)
None	14382 (63.8)
Some	5363 (24.5)
All	2541 (11.7)

TABLE S5. Associations between having identity documents with one's preferred gender marker and psychological distress

Identity Documents Reflect Gender	Unadjusted *b* (95% CI)	Adjusted *b** (95% CI)	Unadjusted PR† (95% CI)	Adjusted PR*† (95% CI)
None	--	--	1.00	1.00
Some	-3.08 (-3.33, -2.82)	-0.59 (-0.84, -0.34)	0.56 (0.53, 0.60)	0.87 (0.81, 0.93)
All	-4.66 (-5.00, -4.32)	-1.64 (-1.98, -1.31)	0.42 (0.38, 0.47)	0.71 (0.63, 0.78)

* Adjusted for age, gender, race/ethnicity, education, poverty, census region, US-born, functional disability, active military duty, medical transition status, family support, and years living full-time in gender.

† Prevalence ratio for serious psychological distress (Kessler 6 ≧ 13)

TABLE S6. Associations between having identity documents with one's preferred gender marker and suicide risk

Identity Documents Reflect Gender	Suicidal ideation		Suicide plan		Suicide attempt	
	Unadjusted PR* (95% CI)	Adjusted PR*† (95% CI)	Unadjusted PR* (95% CI)	Adjusted PR*† (95% CI)	Unadjusted PR* (95% CI)	Adjusted PR*† (95% CI)
None	1.00	1.00	1.00	1.00	1.00	1.00
Some	0.72 (0.69, 0.76)	0.95 (0.90, 0.99)	0.73 (0.68, 0.79)	1.01 (0.91, 1.11)	0.76 (0.64, 0.90)	1.14 (0.95, 1.37)
All	0.56 (0.52, 0.60)	0.80 (0.74, 0.87)	0.52 (0.45, 0.59)	0.81 (0.70, 0.93)	0.59 (0.45, 0.75)	1.14 (0.87, 1.49)

* Prevalence Ratio

† Adjusted for age, gender, race/ethnicity, education, poverty, census region, US-born, functional disability, active military duty, medical transition status, family support, and years living full-time in gender.
